# Mapping an EPA-based comprehensive curricular proposal for UME

**DOI:** 10.15694/mep.2019.000186.1

**Published:** 2019-10-07

**Authors:** Alicia Hamui-Sutton, Tania Vives-Varela, Verónica Daniela Durán-Pérez, Samuel Eloy Gutiérrez-Barreto, Manuel Millán-Hernández

**Affiliations:** 1 Faculty of Medicine

**Keywords:** Curriculum, Entrustable Professional Activities, Educational model, Competencies, Competency-based medical education.

## Abstract

This article was migrated. The article was marked as recommended.

In medical education, there have been three important reforms: science-based curriculum, problem-based learning, and competency-based education. Currently, the concept of Entrustable Professional Activities (EPA) goes a step beyond competencies. The aim of this paper is twofold: first, to present a proposal for an EPA-based curriculum for undergraduate medical education, and second, to describe its curricular framework, educational model and organization. This curricular proposal integrates EPA-based education with the foundations of the interpretive epistemology, the constructivist paradigm and the health care transformations. Using Actividades Profesionales Confiables (APROC) as a curricular guideline helps the educators define knowledge, skills, and attitudes; teachers can plan activities that link theory to practice; evaluators can assess the student’s performance and provide him with feedback, and Faculty leaders and collaborators can implement projects to improve educational quality. The curriculum proposal includes a flexible modular system, the integration of biomedical, socio-medical and clinical sciences, and a close link between theory and practice. This curriculum puts medical education at the forefront, it favors the comprehensive education of future physicians, and it constitutes a true educational revolution.

## Introduction

In medical education, there have been three important reforms. First, at the beginning of the 20 ^th^ century, with science-based curriculum-guided teaching, the curriculums were divided into subjects and there was a clear limit between “basic” and “clinical” sciences. Mid-20 ^th^ century, some schools implemented the problem-based learning (PBL) approach. Late in the 20 ^th^ century, medical schools adopted the flexible curricular models of competency-based education (CBE) ( Morales Castillo and Varela Ruiz, 2015). Currently, the concept of entrustable professional activities (EPA) is a proposal that helps to make a competency-based curriculum operational (ten Cate, 2005).

The aim of this paper is twofold: first, to present a proposal for an EPA-based curriculum for undergraduate medical education (UME), and second, to describe its curricular framework, educational model and organization.

### Undergraduate medical education: classical organization

Many medical schools have a classical course of studies which maintain the Flexnerian legacy, organized as two years of preclinical basic sciences, two years of clinical courses, one year for the undergraduate internship, and one year of Social Service. Few schools have a modular organization.

Medical education is heterogeneous because it is organized by institutions with marked differences in their development as well as in their educational offering. In addition, schools and faculties can be public or private. AMFEM (Mexican Association of Medical Schools and Faculties) classifies them into two groups. The first includes schools aligned with a formation clearly directed towards the specialization of their graduates; considering the medical degree as a kind of introductory course for a later specialization. The other group is formed by schools aimed to train general practitioners, seeking to answer the health requirements of society; this group faces limitations due to a poor labor market that offers more places for specialists than for general practitioners (AMFEM, no date).

In other hand, the curricula of some schools and faculties have not changed during the last 5, 10, 15 or even 20 years. Therefore, the response to health needs is heterogeneous and not always consistent with changes in the national context. The international trend to address this situation has been the creation of competency-based curricula.

### Justification

Studies have been conducted regarding the implementation of competency-based education ( Hamui-Sutton, 2015). Some of the findings were:

• The career induction is insufficient.
• The subjects suffer from a rift between theory/practice and basics/clinical.
• There is a fragmentation of knowledge in subjects and a duplication of contents.
• The academic programs have an overload of specialized contents.
• There are high rates of academic failure and desertion.
• Competencies are difficult to make operational.
• The curriculum, student trajectory, career choices, and professional plan are inflexible.
• The formation does not integrate the student’s social and cultural development with his/her personal life project.

There are many causes for the difficulty of making competencies operational: 1) the definition and comprehension of competencies is complex, 2) there is confusion between learning, competency, and performance, 3) the comfort zone of continuing with outcome-based learning and summative assessment and, 4) the prevalence of educational strategies centered on the teacher. These elements unbalance the competency-based curriculum within the educational process ( Hamui-Sutton, 2015).

## Theoretical framework

### The EPAs as an educational model

As multidisciplinary team of researchers in medical education, we design a model to make competencies compatible with the educational process and make them operational. After reviewing national and international proposals of competencies, the team concluded that competencies are abstract, and difficult to teach and to assess. Then, the research team performed a qualitative study with focus groups to identify the activities/competencies that a general physician uses after UME ( Hamui-Sutton *et al.*, 2015a, 2017).

In 2014, the Association of the American Medical College (AAMC) published the “Core Entrustable Professional Activities for Entering Residency” which contains 13 Entrustable Professional Activities (EPA) ( AAMC, 2014). The concept of EPAs began in 2005 (ten Cate, 2005) and it became an international trend in 2010. Many countries tried to apply EPAs, but the projects were centered in medical specializations, internship or the transitional period.

After an analysis, our UME project integrated the EPAs to the proposal as a central part. Using EPAs is a way to make competencies operational because it is a precise task that requires many competencies (ten Cate, 2013). It helps the educators to define knowledge, skills, and attitudes; teachers can plan activities that link theory to practice; evaluators can assess and provide feedback of the student’s performance, and Faculty leaders and collaborators can implement projects to improve educational quality.

With the integration of the EPAs, we developed different projects, these are the key facts ( Hamui-Sutton *et al.*, 2018):

1. Contextualization of the concept of EPAs as Actividades Profesionales Confiables (APROC) ( Hamui-Sutton *et al.*, 2015b).
2. Design of the CARAIPER scheme, an educational strategy for the learning and teaching of clinical reasoning ( Durán-Pérez, 2019), and the construction of lesson plans called “cédulas” ( Torruco-García *et al.*, 2016).
3. Creation of specific APROCs ( Hamui-Sutton *et al.*, 2017) and their contextualization within the regulations and characteristics of our country ( Gutiérrez-Barreto *et al.*, 2018).
4. Development and implementation of an assessment system for the undergraduate internship ( Hamui-Sutton *et al.*, 2017, 2018).
5. Planning, implementation, and evaluation of educational projects in various subjects: Gynecology & Obstetrics, Health Promotion, Biomedical Informatics, Anatomy, Pharmacology, Surgery and Embryology ( Hamui-Sutton *et al.*, 2018).

To proceed into the transformation of medical education, the curricular proposal integrates EPA-based education and the foundations of ( Hamui-Sutton *et al.*, 2018):

• the ***interpretative epistemology*** that supports the idea that theory is interpreted in the light of experience in concrete contexts, with the organizational, social and personal elements in which practice and reflection take place.
• the ***constructivist paradigm*** which considers that reality is socially constituted and is based on experience. It assumes that knowledge is generated by interaction and interpretation. The four main pedagogical foundations are: experiential and situated learning, and reflective and deliberate practice.
• Also, ***health care transformations*** pose challenges and require medical education to improve the training of health professionals and to standardize educational quality. It is important to consider the context of the current exercise of medicine to design and implement strategies that favor the personal and professional development of the future physician.

### Curriculum Basics

In this paper, we present a curricular proposal that integrates three elements: curricular framework, educational model and intended curriculum.

Defining a curricular framework is complex. It is the expression of an epistemological vision and a work method which considers the participants and the social interactions within the educational process. It is the central idea of the educational model and it is at the core of all the institution’s processes related to learning: selection, organization, distribution, transmission, and assessment of contents.

Each curriculum includes an educational model. It is a conceptual network of pedagogical theories and approaches which guides the design of the course of study and which systematizes the educational process. Educational models vary according to the historical moment. Their validity and usefulness depend on the social, scientific, and technological context. An intended curriculum is the set of all teaching/learning experiences that must be studied to obtain a degree. It involves the selection of contents required to achieve objectives, how to approach structure and organization, their importance and the timing of learning ( Díaz-Barriga, 1997).

Once the learning contents are selected, it is mandatory to organize and distribute them among the various courses of the curriculum. They are distributed progressively, cyclically or concentrically, but in all cases, a horizontal and vertical continuity with the level of the course contents must be assured.

## EPA-based Curriculum

As background, we want to mention the Utrecht EPA-based undergraduate curriculum ( ten Cate *et al.*, 2018), because that work has similarities and differences with ours and it represents a great step forward. It has five core EPAs with 31 smaller nested EPAs which guide the curriculum. It transforms the organization of clinical education through the creation of five LINK blocks. Each one combines from three to six medical specialties within the logic of longitudinal integrated clerkship (LIC) ( Hirsh, Holmboe and ten Cate, 2014).

**Figure 1.**
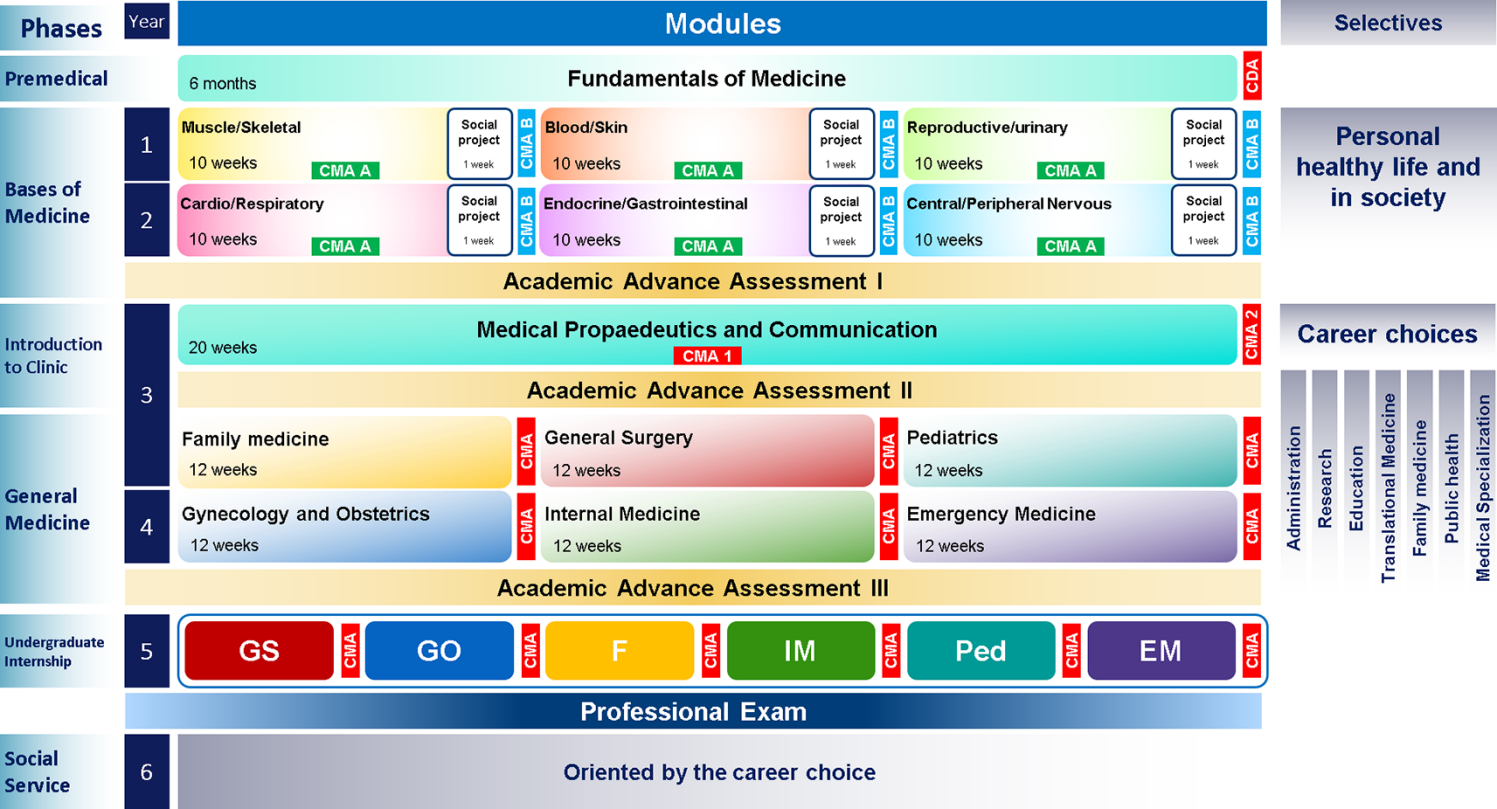
EPA-based Curriculum Map for UME

After analyzing the Utrecht proposal, we designed our EPA-based curriculum for UME, ( Figure 1). It has three levels: macro-, meso-, and micro-.

### MACRO-level

The first element of the macro level is the ***flexible modular system***. A module organizes the contents in complete learning units, with a holistic and comprehensive vision. It offers flexibility because each student draws from his/her own academic trajectory until finishing the UME ( Akbhsl-García and Ortega-Esparza, 2006).

The modular system follows the curricular principles, which define the educational goals and design of the curriculum, and which interweave in order to interconnect the contents using an interdisciplinary approach. They guide the education according to social demands, international trends, scientific and technological advances, and the graduate profile. ***The APROC are the curricular principles***, which focus the educational process on general medical practice.

The six General APROC define the ***graduate profile***. They are the activities that the general physician performs because he/she is competent and able to provide healthcare with professionalism, safety, and quality. The six APROC are:

G1. To provide comprehensive medical care to the stable patient

G2. To perform medical procedures to treat health events

G3. To interact with the patient and his/her family in order to share the decision-making

G4. To carry out health promotion and disease prevention actions according to the policies of the National Health System

G5. To collaborate with interdisciplinary teams for patient care

G6. To provide initial emergency care to the unstable patient

The acquisition, development and performance of each APROC requires continuous interaction between patients and community. Each APROC requires and combines different competencies. It uses the AAMC’s framework, which has eight domains of competence ( Englander *et al.*, 2013).

In order to maintain consistency between the modular system and the APROC, the assessment must be holistic. To this end, we established the Comprehensive Modular Assessments (CMA), the Collegiate Evaluation (CE) and the Academic Advance Assessments (AAA) ( Figure 1). The final grade of a module consists of 50% CMA grade and 50% CE. The CMA are theoretical exams by competencies and APROC. They are designed using the cognitive analysis of tasks ( Peñalosa and Castañeda, 2009). The CE is the grade agreed upon by the teachers of the module for each student. After passing all the modules of each phase, the student presents an AAA. It is a diagnostic, formative, and theoretical/practical evaluation that provides feedback about the student’s reliability, performance and competency levels in APROC and competencies.

In our country, most physicians are in general practice or primary care, but society needs physicians to get involved in other fields, like research, education, public health, healthcare management.. that is why we established the ***Career Choices***, in addition to the graduate profile, for professional development after graduation.

A way to encourage the development of the APROC and competencies, while at the same time supporting the ***personal and professional trajectory of the student***, is achieved by establishing a close relationship with students. To promote this bond, we considered the formation of medium-size groups (20 students maximum), and the redesign of the tutorship program. Each group will be handled by four to eight teachers to ensure interdisciplinarity and a tutor who will support them throughout their undergraduate studies. The tutor assists with conflicts within the educational process, he/she orients and refers his/her students if they need specialized help (academic, psychological, ..) ( Calkins and Epstein, 1994; Fresko, 1996; Kalén *et al.*, 2010; Steele, Fisman and Davidson, 2013; Ghenghesh, 2018).

Finally, the ***curricular assessment*** needs to include qualitative and quantitative approaches in order to analyze the experience of students, teachers, and academic and administrative staff, because this curriculum involves the transformation of all educational processes. Such innovation also requires people to take responsibility for changes and a commitment to them. The assessment must be continuous, and its results are useful for decision-making regarding educational projects and planning strategies to improve the adaptation process.

All elements of the macro-level of the curriculum are designed to integrate biomedical, socio-medical and clinical learning, and to promote the link between theory and practice through a critical reflection which interprets them as closely related parts (Schwartzman, Roni y Eder, 2013). This favors an interdisciplinary vision and the application of knowledge in concrete reality. It also requires teacher training, educational research, and technological innovation.

### MESO-level

The macro-level influences and guides the meso-level, which is comprised of the curricular map and its organizational criteria (phases, modules, and electives). The curriculum lasts six years plus one more for social service. It is divided into six phases:

***First phase: “Premedical”:*** it includes the “Fundamentals of Medicine” module, which lasts six months. This phase aims to homogenize core knowledge and competencies before beginning UME. The module combines the introductory topics of all disciplines of the second phase. For example, the student reviews planimetry, -a basic theme of anatomy-, microscopy for histology or microbiology.. It also provides orientation regarding physicians’ personal and professional lives; this reflection aims at reinforcing whether the student really wants to dedicate his or her life to medicine. If not, the student can select another professional path.

**Figure 2.**
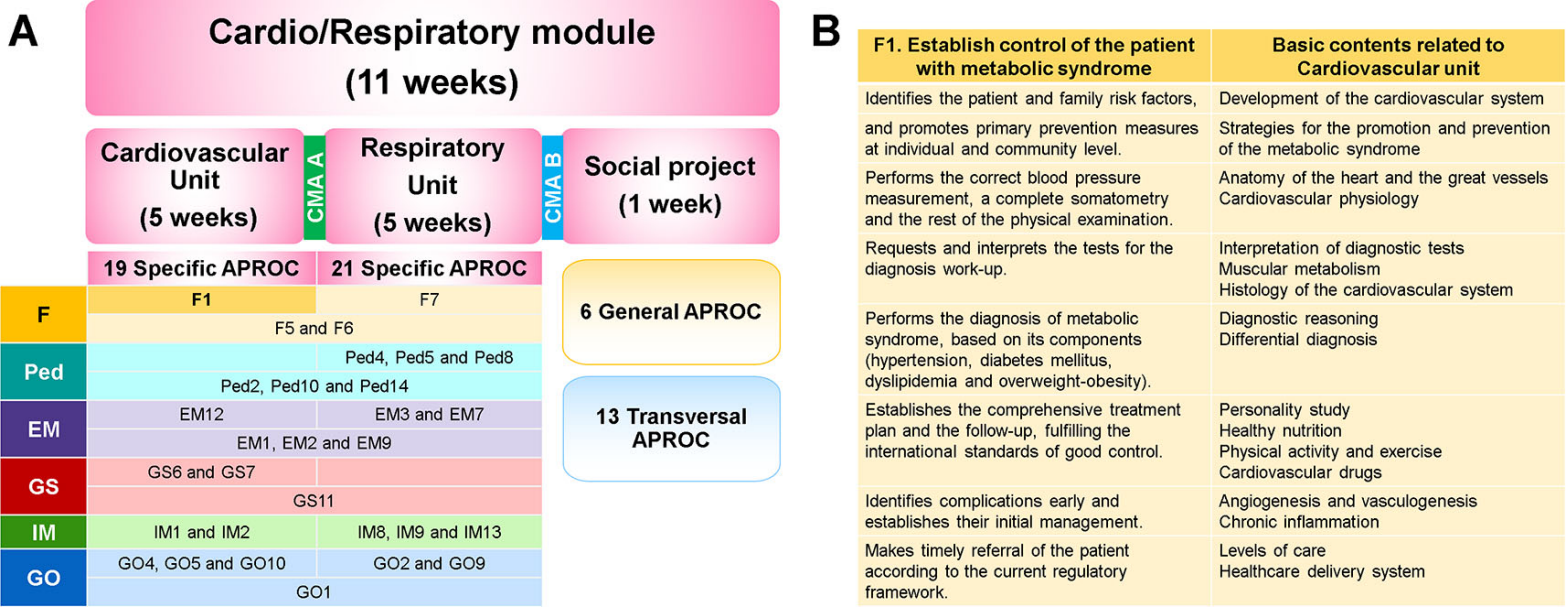
A module of the Second phase, “Foundations of Medicine”

***Second phase: “Foundations of Medicine”:*** this phase lasts two years. Students attend classes in the educational institution and in the primary care units. It includes six modules, each one lasting 11 weeks, and it follows a systems approach and a group of APROC which determines the biomedical, socio-medical and clinical contents. The first-year students choose the sequence of three modules: muscle/skeletal, blood/skin and reproductive/urinary; and on the second year, they choose from among three other modules: cardio/respiratory, endocrine/gastrointestinal and central/peripheral nervous system. In Figure 2A, we can exemplify the relationship between APROC and the contents and to explain the organization thereof.

Now the question is: How to identify the contents? One method is: 1) select an APROC, 2) review its description, 3) analyze the associated teachings and, 4) establish what are its basic contents of biomedical, socio-medical and clinical sciences ( Figure 2B). During lessons, teachers work together to ensure the interdisciplinary vision of the contents and to integrate knowledge ( Harden, 2000; Brauer and Ferguson, 2015; Mookerjee *et al.*, 2018).

A strategy to improve the theoretical and practical relationship, and the bond with community health, is the application of a Social Project at the end of each module. The project is designed during the previous ten weeks and integrates the contents related to a community need -for example, the timely detection of hypertension and the promotion of healthy habits ( Moses, 1987; Laing and Howell, 1994; Barss *et al.*, 2008; Dharamsi *et al.*, 2010).

**Figure 3.**
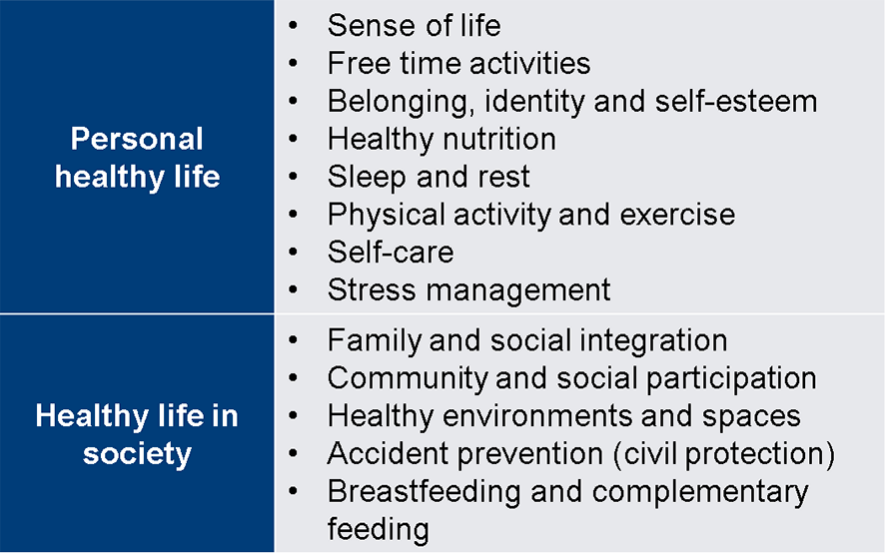
Electives of “Personal healthy life and healthy life in society”

With the aim of promoting the students’ quality of life and well-being, we added the elective subjects called “Personal healthy life and healthy life in society” ( Figure 3). They award mandatory credits and the student chooses to study one in parallel with each module of the second phase ( Barss *et al.*, 2008; Rakel and Hedgecock, 2008; Mookerjee *et al.*, 2018).

**Figure 4.**
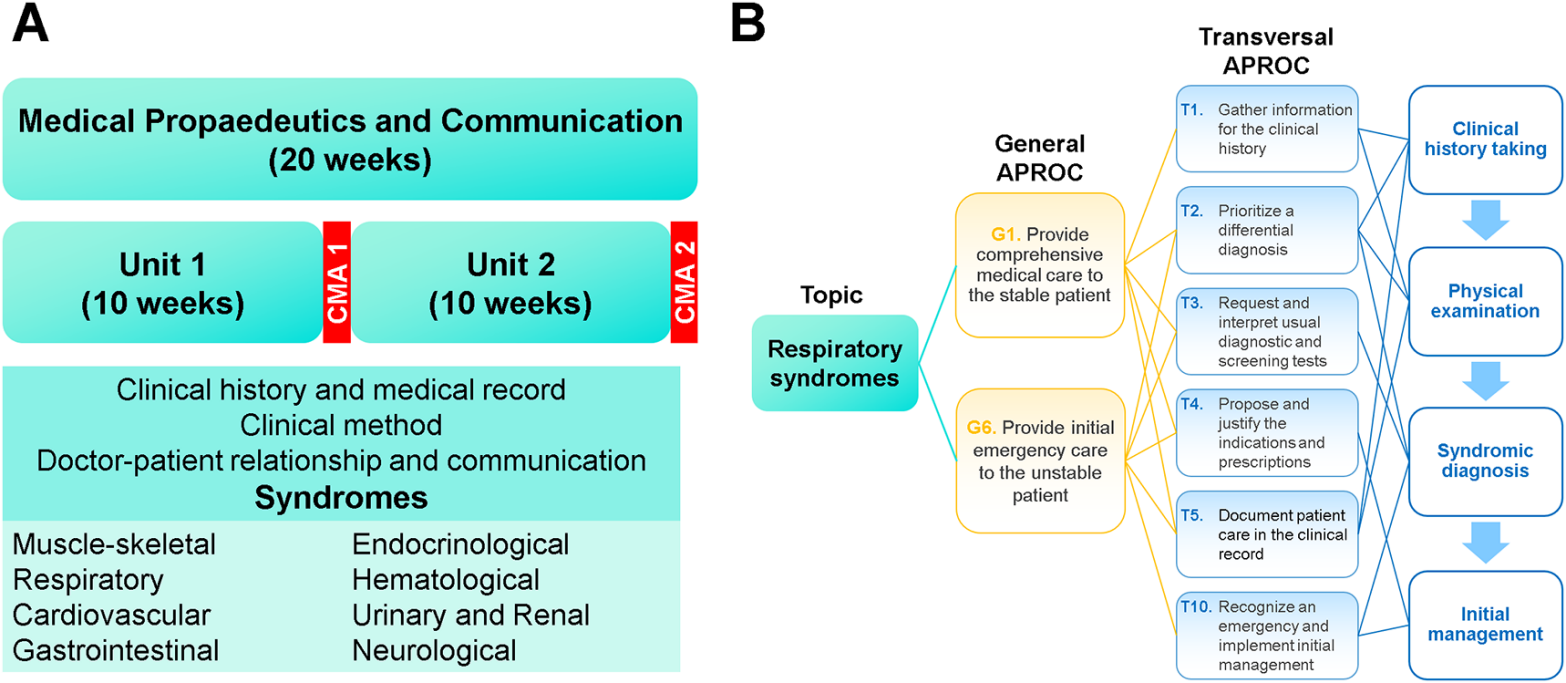
Module “Medical Propaedeutic and Communication” of the Third phase “Introduction to Clinical Practice”

***Third phase: “Introduction to Clinical Practice”:*** it offers the module “Medical Propaedeutic and Communication”, which lasts 20 weeks and is opened twice a year ( Figure 4A). If a student fails a module of the second phase, he/she has a second chance to re-take the module and enters the third phase one semester later. The module has two units of 10 weeks each. During this period, students attend lessons and practice in primary care clinics.

The organization of contents follows a logical sequence of learning: clinical history, physical examination, syndromic diagnosis and initial management ( Figure 4B). At the end of the third phase, students participate in an orientation fair to get information about the Career Choices, and then each student select one (right side of Figure 1).

**Figure 5.**
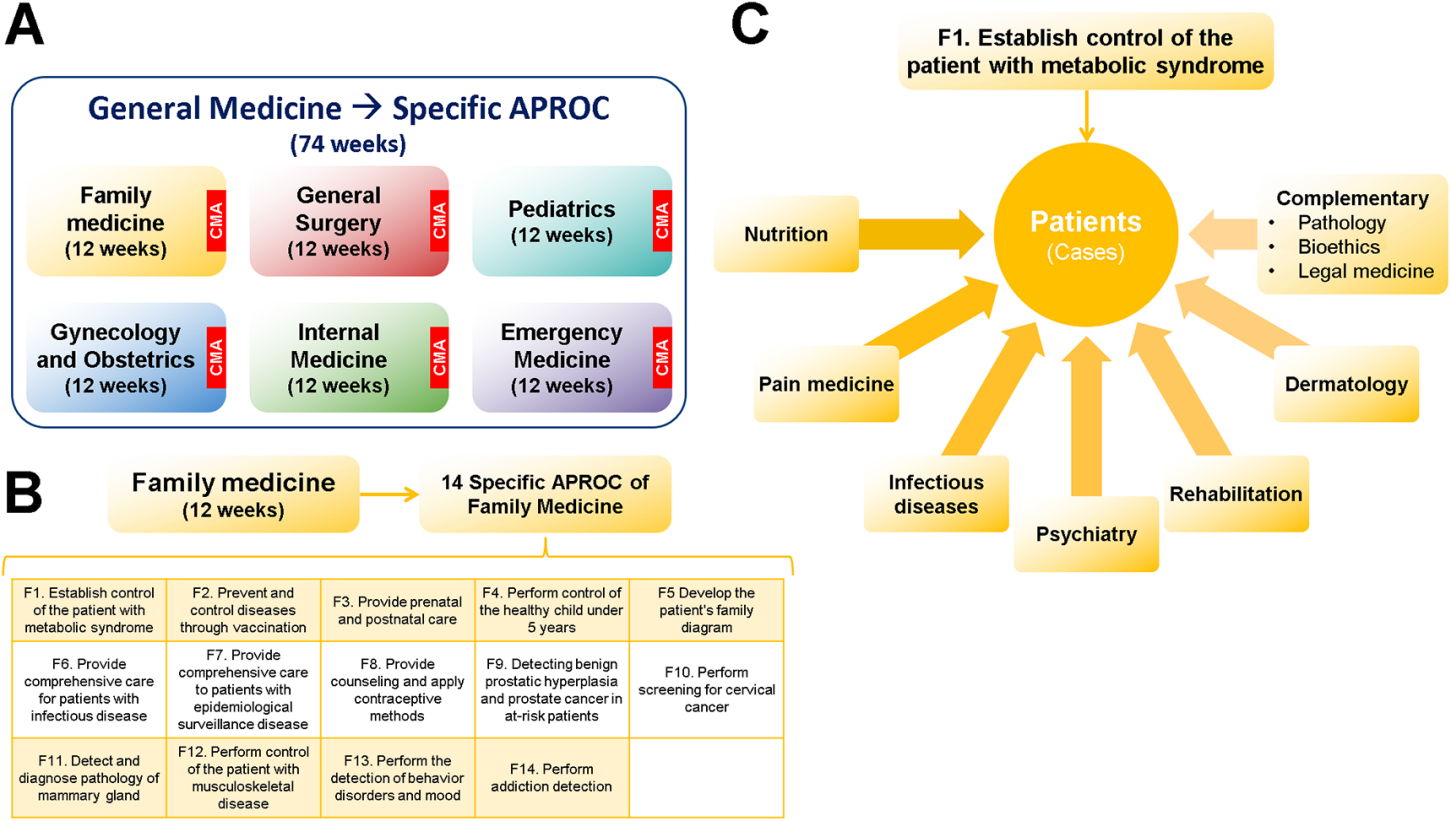
Fourth phase “General Medicine”

***Fourth phase: “General Medicine”:*** this phase has six modules. The entire phase lasts 74 weeks: each module lasts 12 weeks and 2 weeks are used for assessment ( Figure 5A). It requires many care units because students must attend clerkships and rounds for the acquisition and development of the specific APROC associated with each module ( Figure 5B).

The fourth phase uses the LICs logic ( Hirsh, Holmboe and ten Cate, 2014). Each module groups disciplines by affinity of content to balance the clinical training of a general practitioner and to favor interdisciplinary patient care. Another reason is that students can rotate through the clinical settings for longer to promote the continuity and integration of the healthcare process ( Figure 5C). Therefore, the modules correspond to the six core medical areas ( Figure 5A). The student chooses the sequence he prefers. For example, a student can start with Family Medicine and another one with Pediatrics. A clinical teacher assumes the responsibility for the module, and he/she can invite specialists to enrich the educational experience. Simultaneously, students take a course from the career choice they selected ( Figure 1). Therefore, at the end of this phase, students have taken 6 courses.

***Fifth phase: “Undergraduate internship” (UI):*** it lasts one year. It is transcendental because the student assumes the role of a medical intern and participates with a health team and in individual and collective healthcare. This experience requires the student to apply the knowledge of previous phases. It promotes the consolidation of the specific APROC and the reinforcement of the general and transversal APROC. The UI has the rotation-based clerkship logic with six rotations, each one lasting two months ( Figure 1). The assessment of each rotation of UI has two elements: CMA and evaluation of clinical practice. The grade combines the judgment of the rotation’s clinical teacher and the result of APROC’s assessing system. At the end of UI, the student must take the professional exam.

***Professional exam:*** this process is an important step before Social Service. The Technical Council of the FM agrees that: “To perform the Social Service, the students of the medical degree [..] must, in addition to passing all subjects established in the curriculum, to have previously passed his/her professional exam” ( Faculty of Medicine, 2005a, 2005b).

***Sixth phase: “Social Service”:*** this phase is regulated by educational institutions and the Secretariat of Health ( Mazón Ramírez, 2012; Graue and Ruiz, 2015). In regulations, Social Service is defined as a temporal and paid job that interns perform for society and the State ( Secretariat of Health, 2014). In this phase, the intern applies his/her learning experience to a field/job related to the career choice that he/she selected. The Career Choices’ offer covers some of the possible professional paths for the general practitioner. If the student so chooses, he/she can complement it with a post-graduate diploma to broaden and deepen his/her expertise. Only if the student completes all credits and requirements for graduation, he/she receives a professional license which will allow him/her to establish a private practice or work in a public institution.

### MICRO-level

The micro-level includes strategies applied to the educational process. We implemented educational projects based on the study of patients (cases). Medical educators must understand the clinical method, medical reasoning and cognitive processes to innovate in education and to design activities focused on the development of competencies and APROC.

From the first to the fourth phases, a learning-teaching strategy called the CARAIPER scheme was created to strengthen the acquisition of clinical reasoning, which is essential for the study and resolution of cases in the health sciences. CARAIPER is the acronym for the following steps: case, clarification of terms, representation of the activity, analysis, integration, questions, independent study, and feedback ( Durán-Pérez, 2019). This scheme is an integral, systematic and flexible strategy, designed with the aim of innovating and improving education through didactic planning. Its flexibility allows the adaptation of the scheme to each module, if it is necessary to include different or specific didactic resources (models, applications, simulators, ..) and sometimes learning contents have their own strategies (role-playing, video, audio, ..).

If learning and teaching change their paradigms, the assessment must be transformed. The educational model prioritizes the learning process and the application of knowledge, that turns the focus to formative assessment and feedback, without neglecting the summative evaluation. With this idea in mind, an assessment system for the fifth phase was created, because it is there that students apply what they learned in a real-life context with living patients, and it is possible to observe and assess their performance on clinical tasks. The assessment system uses an app to identify and register the expertise level of each student. The assessment of each APROC begins with the student, in order to promote a metacognitive process about his/her advance on clinical performance. Afterwards, it is important for a clinical teacher, other physicians, and residents to assess the students, as they, as clinical experts, can provide feedback about how to improve and what to correct.

## Conclusions

The curriculum applies these medical principles: “Treat the patient, not just the disease!”, “To be a great physician, you must understand the whole story”, “Sometimes to cure, often to relieve and always to comfort” and “Each patient is a unique human being, not a disease or a group of symptoms”. These aphorisms demand acting on medical education, emphasizing the integration and balance between three principles: science, humanism, and society, because a general physician must be reliable in any task where he/she provides care.

These priorities must be reflected and combined in the educational process because a student is not a black box where contents are introduced and then the student magically turns into a physician. Therefore, all medical schools are responsible for the training of competent health professionals, because they will be responsible for providing safe, comprehensive and quality care to individuals and populations.

A way to achieve this goal is the curricular design of better UME programs, applying educational trends that are consistent with current needs. It is in this manner that the APROC trend goes a step beyond competencies and becomes a true educational revolution. When APROC is the axis of a curriculum, it affects each level of application (macro-, meso-, and micro-), causing changes in all phases of the educational process. The curriculum is a proposal to put medical education at the forefront and to fulfill our mission as educators, favoring the comprehensive education of future physicians.

## Take Home Messages

• The APROC (or EPAs) as a curricular axis goes a step beyond competencies and becomes a true educational revolution.
• Medical schools interested in applying EPAs could implement this curriculum.
• Applying an educational model requires consistency and changes to the curricular design, educational process, and student-teacher relationship.
• This curricular proposal includes a flexible modular system, the integration of biomedical, socio-medical and clinical sciences, and a close theoretical-practical bond.
• This EPA-based curriculum puts the medical education at the forefront and favors the comprehensive education of future physicians.

## Notes On Contributors

**Alicia Hamui-Sutton:** Sociologist, MSc, PhD in Social Sciences. Head of the Secretariat of Medical Education and Professor at the Faculty of Medicine of the National Autonomous University of Mexico (UNAM). Named National Researcher on medical education and health anthropology. Member of the National Academy of Medicine of Mexico. ORCID iD: https://orcid.org/0000-0002-3190-4470

**Tania Vives-Varela:** Doctor of Sciences in the disciplinary field of Education in Health Sciences of the field of knowledge of Sociomedical Sciences. Master’s degree in Communication and Educational Technology. Degree in Psychology. Professor at the undergraduate and graduate level. Head of the Department of Research in Medical Education of the Secretariat of Medical Education at the Faculty of Medicine of the UNAM.

**Verónica Daniela Durán-Pérez:** Master of Education in the Educational Management Area at La Salle University. Medical Degree, Professor of Biomedical Informatics and Health Promotion, Collaborator of the Department of Research in Medical Education and collaborator of the Academic Development Unit at the Faculty of Medicine of the UNAM. ORCID iD: https://orcid.org/0000-0002-0901-2724

**Samuel Eloy Gutiérrez-Barreto:** Master of Sciences in the disciplinary field of Education in Health Sciences of the field of knowledge of Sociomedical Sciences. Medical degree. Collaborator of the Department of Research in Medical Education of the Secretariat of Medical Education and Professor at the Faculty of Medicine of the UNAM.

**Manuel Millán-Hernández:** Master’s degree in Directive Management in Health. Medicine Specialist in Family Medicine. Medical degree. Medical doctor and Professor in the Department of Emergency Medicine at the Mexican Social Security Institute. Collaborator of the Department of Research in Medical Education of the Secretariat of Medical Education and Professor at the Faculty of Medicine of the UNAM. Member of Mexican Society of Anatomy.
