# Supplementary material for: Mapping an EPA-based comprehensive curricular proposal for UME
Source: MedEdPublish (2016). 2019 Oct 7;8:186. [Version 1] doi: 10.15694/mep.2019.000186.1 (PMC10712502; doi:10.15694/mep.2019.000186.1)
Supplement: Peer review history

# Reviewer report: Reviewer response for version 1

**DOI:** 10.21956/mep.19906.r30146

**Published:** 2020-01-04

**Authors:** Ken Masters

**Affiliations:** 1 Sultan Qaboos University

This review has been migrated. The reviewer awarded 4 stars out of 5 An interesting article dealing with mapping an EPA-based comprehensive curricular proposal for UME. The authors have outlined, in some detail, their view of the curriculum. While the plan is not revolutionary, it does provide a solid base on which to build courses.I am, though, a little concerned about Phase 1. (Let me begin by saying that my concern may simply be that not enough detail has been supplied, so perhaps the authors could supply some more detail):• The name is "Fundamentals of Medicine" / "Bases of Medicine". In the topics, though, I see only really Human Biology (and then some “Selectives,” but very much on the side). While knowledge of Human Biology is an important base of Medicine, it is one only. So, I would recommend that one or more of the following be done to the model:o The stage be renamed to Human Biology, and / oro Details of the individual components be given so that it is obvious that they go far beyond Human Biological issues, and / oro Some of the other bases of medicine be included directly and specifically so that the broad naming is appropriate.• In the Introduction, the authors refer to the weaknesses of subject-based teaching, and the value of PBL in overcoming this. Parts of the philosophy shown in this paper encourage integration, but the diagram breaks the basics into strictly-defined, subject-based silos, and there is the very real danger that, in practice, it will inevitably lead to an implementation in which the worst fears of isolation-teaching, repetition and gaps occur. I would recommend that the authors describe in detail how they plan to avoid these. This detail should be more than merely a statement of “constant review”, as many medical schools have this belief, but it usually translates into infrequent meetings by ill-constituted committees, staffed by people who have far more important clinical and other concerns than those of following up on possible repetition and gaps across courses. So, the paper is a useful description of what has been envisaged; I do think, though, that the authors need to address some issues plainly before implementation.

Reviewer Expertise:

NA

No decision status is available

# Reviewer report: Reviewer response for version 1

**DOI:** 10.21956/mep.19906.r30145

**Published:** 2019-10-13

**Authors:** Subha Ramani

**Affiliations:** 1 Harvard Medical School, Brigham and Women's Hospital

This review has been migrated. The reviewer awarded 4 stars out of 5 I commend the authors for doing such a comprehensive description of the 3 main traditions of science-based, problem-based and competency-based education. Their description of each model and downstream healthcare effects is very clear and well argued.The aims of this paper are clearly stated. I like their description of their curricular proposal integrating the 3 elements of curricular framework, educational model and intended curriculum. They describe the process of developing their APROCs clearly.I completely agree with their conclusion that the patient and not the disease should be treated and curriculum and assessment should be holistic in nature.The graphic representation of their curriculum is very good.I am confused about the theoretical foundations talking about the epistemology, constructivist paradigm and health care transformations.Looking at their curriculum at various levels, macro, meso and micro makes sense to emphasize different curriculum elements. However, the descriptions were too detailed and sometimes hard to follow.Clearly, there is a lot of expertise on the team and a lot of valuable information in this paper, but keeping it more concise with increased clarity in the structure of the sentences and a road map for readers to know where they are being led would be very useful.The paper will be useful to educators engaged in assessment systems using EPAs.

Reviewer Expertise:

NA

No decision status is available

# Reviewer report: Reviewer response for version 1

**DOI:** 10.21956/mep.19906.r30144

**Published:** 2019-10-09

**Authors:** Felix Silwimba

**Affiliations:** 1 University of Lusaka

This review has been migrated. The reviewer awarded 5 stars out of 5 clearly articulated innovative medical education approach. I recommend for all medical educators

Reviewer Expertise:

NA

No decision status is available
